# Neural Correlates of Cognitive Gains Induced by Commercially Available Cognitive Training Programs: A Meta-Analysis of Neuroimaging Studies

**DOI:** 10.3390/brainsci16010078

**Published:** 2026-01-06

**Authors:** Ziqin Wang, Yang Liu, Chengzhen Liu, Geng Li

**Affiliations:** 1 School of Psychology, Research Center for Exercise and Brain Science, Shanghai University of Sport, Shanghai 200438, China; 2 School of Sports Science, Jishou University, Jishou 416000, China; 3 Faculty of Psychology, Southwest University, Chongqing 400715, China

**Keywords:** cognitive training, commercial cognitive programs, brain activation, meta-analysis

## Abstract

**Background**: Commercial cognitive training programs are widely marketed as tools for enhancing cognitive performance, yet training-related task-related brain activation changes remain incompletely characterized. This preregistered meta-analysis aimed to synthesize evidence on whether commercially available cognitive training is associated with improvements in cognitive function and convergent alterations in task-related brain activation, and to explore factors that may moderate these effects. **Methods**: A multivariate meta-analysis was conducted on behavioral outcomes to estimate the overall effect of training on cognitive performance. Task-based neuroimaging findings were synthesized using a coordinate-based neuroimaging meta-analysis to identify consistent activation changes associated with training. Exploratory analyses examined whether participant characteristics and training parameters were associated with training-related activation changes and whether these changes were related to cognitive improvement. **Results**: Commercial cognitive training was associated with a significant moderate improvement in cognitive performance (Hedges’ g = 0.485; 95% CI = 0.149–0.821; t = 2.924; *p* = 0.006). Neuroimaging analyses revealed increased activation in the left anterior cingulate cortex (L.ACC), right inferior frontal gyrus (R.IFG) and right superior temporal gyrus (R.STG), together with decreased activation in the right supplementary motor area (R.SMA). In exploratory analyses, training frequency, compliance and age were associated with differences in training-related brain activation. Activation within the L.ACC and R.IFG was significantly related to cognitive improvement. **Conclusions**: Commercial cognitive training was associated with cognitive gains and convergent task-related activation differences across studies. These findings provide the first quantitative neuroimaging synthesis of commercial cognitive training and highlight training frequency, compliance and age as potential moderators of training-related neural outcomes.

## 1. Introduction

Cognitive function refers to the set of mental processes that support learning, reasoning and self-regulation, and it forms the foundation for everyday competence, psychological well-being and social adaptability [1,2]. Declines in core processes such as executive function, attention and memory can erode learning efficiency and impair decision-making and behavioral regulation [3]. These changes often reduce independence in daily activities and ultimately affect quality of life and societal participation [4]. Identifying approaches that can preserve or enhance cognitive function is therefore essential [5,6]. Although pharmacological interventions can be beneficial in certain contexts, their broader use may be limited by side-effects, adherence challenges and restricted suitability across populations, underscoring the need for non-pharmacological, low-risk and scalable alternatives [7].

Cognitive training has consequently received increasing attention, given its safety, ease of implementation and capacity for adaptive, ability-based progression [7,8,9,10]. However, although cognitive training is intended to strengthen core cognitive processes, prior work has relied predominantly on highly structured laboratory paradigms [11]. Training has often targeted a single cognitive domain or a single component process, with evaluation focused primarily on performance within the trained task and changes on near-transfer measures [12]. These features can constrain ecological relevance and limit the generalizability of findings, thereby restricting broader real-world application [13,14]. With advances in digital technology, cognitive training has increasingly extended beyond laboratory settings into wider applied contexts, alongside rapid growth in commercially available cognitive training programs [7]. Typically delivered via digital platforms, these programs are widely promoted and used as tools to support cognitive health in everyday life [15]. Compared with traditional laboratory paradigms, commercial programs more commonly adopt modular, multi-domain training frameworks. They can be delivered in non-laboratory settings such as homes and community environments in a relatively standardized manner, and they often incorporate adherence-support features such as automated feedback, ability matching, and staged progression to facilitate sustained, long-term training and follow-up [15].

Multi-domain training approaches also exist in laboratory research, but task selection and training structure are usually tailored to specific theoretical questions, emphasizing experimental control and mechanistic decomposition [12]. As a result, these approaches are not fully equivalent to the “task-library” style assembly that characterizes many commercial suites. Platforms frequently represented in the current literature—such as RehaCom^®^, Cogmed^®^, Lumosity^®^, Happy Neuron Pro^®^, CogPack^®^, and related packages—typically offer menus of brief, repeatable exercises with performance-adaptive difficulty, while individual studies may differ substantially in which modules they select and how they combine them [16,17,18]. For example, RehaCom^®^ is often implemented as a structured set of modules spanning attentional control and executive–memory operations [19], whereas published Cogmed^®^ protocols more commonly configure training around adaptive working-memory practice [18]. CogPack^®^ and other rehabilitation-oriented suites tend to employ broader module combinations that integrate exercises targeting processing speed, attention, memory, and executive control [20]. Overall, the trained processes across commercial platforms commonly include working-memory maintenance and updating, selective and sustained attention and processing speed, inhibitory and interference control, cognitive flexibility, and higher-order problem solving [21,22,23].

Consequently, even when studies nominally use the same commercial platform, differences in module composition and training structure may meaningfully alter the effective cognitive ingredients of the intervention, plausibly contributing to heterogeneity in behavioral outcomes and potentially in neural outcomes as well. This implementation-level variability also provides a plausible context for inconsistent conclusions in prior systematic reviews and meta-analyses, with some reporting cognitive improvements whereas others suggest that overall benefits are modest [7,21,22]. In this context, mechanistic measures may be particularly valuable for testing whether heterogeneous commercial protocols nonetheless yield reproducible patterns of change. Neuroimaging can complement behavioral indicators by providing mechanism-oriented evidence, enabling tests of whether heterogeneous training protocols converge on shared patterns of task-related brain activity and whether such patterns align with cognitive gains [24].

From a task-based mechanistic perspective, different training components may preferentially influence partially dissociable functional networks [25]. Training targeting working-memory maintenance and updating is often linked to modulation of frontoparietal control circuitry [26]; training emphasizing inhibition and conflict monitoring more frequently implicates cingulo-insular systems and inhibitory-related regions, including the right inferior frontal gyrus [27]; and training focused on attention and processing speed is commonly associated with recruitment changes within dorsal attention and related frontoparietal regions [28]. Consistent with this framework, neuroimaging reviews and meta-analyses indicate that working-memory training is associated with changes concentrated in frontoparietal and select subcortical regions, with effects moderated by training duration and task characteristics [29]. Broader syntheses of cognitive training studies further suggest training-specific engagement patterns in medial frontal control-related regions [30]. Importantly, training-related activation changes are not uniformly unidirectional; they may manifest as increased recruitment, consistent with stronger control mobilization, or as reduced activation, consistent with greater processing efficiency [31]. Given that evidence for commercial programs is often anchored in trained-task performance and near-transfer outcomes, focusing on task-state neural activity also facilitates evaluation of correspondence across training, neural modulation, and behavioral change within a shared operational framework.

Despite these advances, the existing neuroimaging evidence base has been derived primarily from laboratory paradigms and relatively domain-specific protocols. It therefore remains insufficient to determine whether commercially available programs—often implemented as multi-domain interventions in non-laboratory settings—produce reproducible task-related activation changes and whether such neural changes are systematically coupled with behavioral gains. Primary studies of commercial programs report mixed directions of activation change. For instance, a digitally delivered EVO^®^ intervention has been linked to altered engagement of cognitive-control circuitry [32]. Cogmed^®^ training has been associated with increased task-evoked activity in frontoparietal regions in some reports [33], but decreased activation in lateral prefrontal and medial frontal areas in others [18]. Accordingly, there is a clear need for a synthesis explicitly focused on commercially available cognitive training to identify whether a convergent common signature of task-related activation change can be detected, and to evaluate heterogeneity through moderator and sensitivity analyses that account for training parameters and participant characteristics. Such an approach can provide converging neural evidence to more precisely delineate the efficacy and mechanistic plausibility of commercially available cognitive training.

## 2. Materials and Methods

In accordance with current best-practice guidelines for neuroimaging meta-analyses [34], we applied both multivariate meta-analysis and seed-based d mapping (SDM) to quantify training-induced changes in cognition and task-related neural activation. Study identification and selection followed the Preferred Reporting Items for Systematic Reviews and Meta-Analyses (PRISMA) framework, with full documentation provided in Supplementary Table S1. The review protocol was prospectively registered in PROSPERO (CRD42024599205). Notably, our final eligibility criteria included an intervention-level refinement relative to the registration: whereas the preregistration did not explicitly restrict the type of cognitive training, the present analysis focused on commercially available cognitive training programs to better reflect their real-world delivery characteristics, accessibility, and implementation sustainability. All other preregistered search procedures, outcomes, and statistical analyses were consistent with the original protocol.

### 2.1. Information Sources, Search Strategy, and Study Selection Process

We systematically searched five electronic databases (PubMed, Web of Science, PsycINFO, MEDLINE, and Embase), with the final update on 12 November 2024. The search strategy followed our prior work [24] and combined controlled vocabulary terms and free-text keywords covering two conceptual domains: (1) neuroimaging and (2) cognitive training. Full search strings for each database are provided in Supplementary Table S2. Reference lists of all eligible studies were manually screened to identify additional records. All retrieved citations were imported into EndNote 21 and deduplicated.

Study selection proceeded in two stages in accordance with PRISMA guidelines. First, titles and abstracts were screened for relevance to cognitive training and functional neuroimaging. Second, full texts were assessed against predefined eligibility criteria. During full-text screening, we verified whether each intervention constituted a commercially available cognitive training program, as required by the present review, and excluded studies using laboratory-developed or noncommercial protocols. Differences in the eligible training paradigms and target populations relative to our prior review [24] were operationalized at this stage through the application of the respective eligibility criteria. All screening steps were conducted independently by two authors (ZW and YL), with discrepancies resolved by discussion or adjudication by a senior author (GL).

### 2.2. Eligibility Criteria

Eligibility criteria were prespecified using the PICOS framework. In alignment with our prior review [24], we retained a broad population scope while adopting a more restrictive intervention definition to address the distinct research question of the present study. Population (P): human participants of any age were eligible, including both healthy and clinical samples. Intervention (I): studies were required to evaluate a commercially available digital cognitive training program, operationalized as an intervention delivered under a named product or platform that was publicly accessible at the time of the study through purchase, subscription, or download [7,21,22]. Custom-built, laboratory-developed, research-only, or otherwise noncommercial protocols were excluded. This criterion represents the primary eligibility distinction from our prior review [32], which primarily synthesized laboratory-developed, single-domain cognitive training paradigms rather than commercially available platforms. Comparator (C): eligible studies included passive control conditions such as waitlist, usual care, or no-training controls, as well as active comparator conditions such as sham cognitive activities, low-challenge control programs, or alternative training controls. Outcomes (O): studies were required to report task-based functional neuroimaging outcomes suitable for coordinate-based meta-analysis, specifically peak activation coordinates derived from whole-brain analyses in standard stereotaxic space (MNI or Talairach). Studies relying exclusively on region-of-interest analyses or without extractable activation foci were excluded. When available, behavioral cognitive outcomes were also extracted to support the multivariate analyses. Study design (S): both within-subject pre–post designs and between-group controlled designs were eligible. Theoretical papers, narrative or systematic reviews, conference abstracts, case reports, and duplicate datasets were excluded.

### 2.3. Study Selection

**Figure 1:**
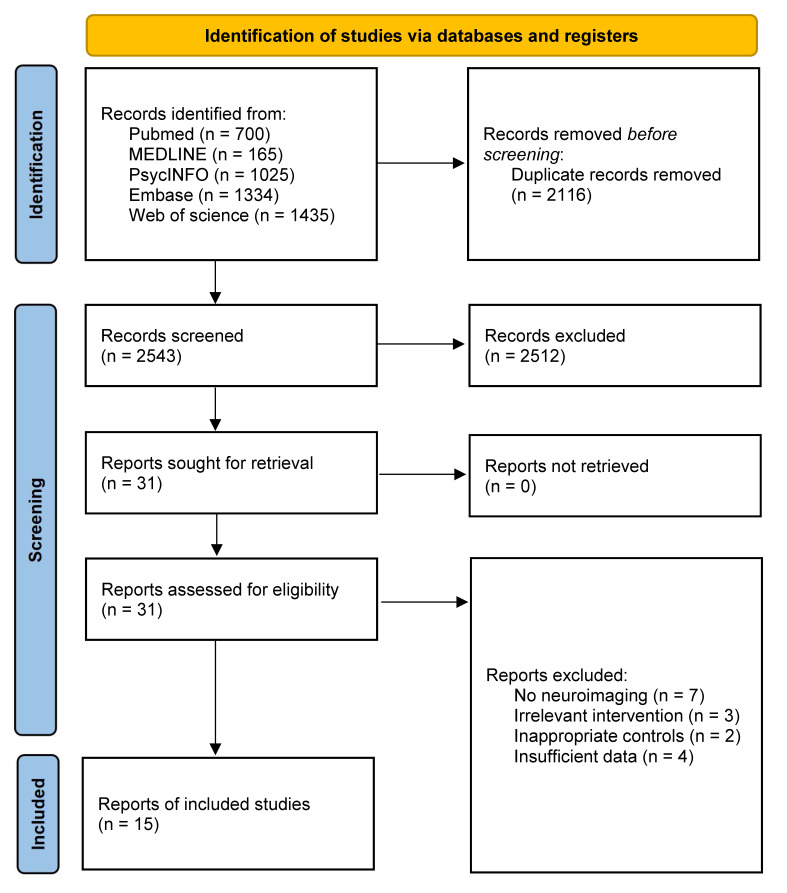
PRISMA flowchart detailing the process for screening and including relevant studies.

To identify studies examining the effects of cognitive training on task-related brain activation, we conducted a comprehensive and systematic search across PubMed, Web of Science, APA PsycINFO, MEDLINE, and Embase using keywords related to neuroimaging and cognitive training. This search yielded a total of 4659 records. After removing 2116 duplicates, 2543 unique records remained. Title and abstract screening resulted in the exclusion of 2512 records that did not meet the inclusion criteria. We then conducted full-text reviews of 31 articles identified through database searches. Ultimately, 15 studies met the eligibility criteria and were included in the final meta-analysis (Figure 1).

### 2.4. Data Extraction and Summary of Outcomes

Two authors independently screened all eligible records and extracted data using standardized templates. Extracted variables included participant characteristics, details of the cognitive training protocol, control group characteristics, and study design features. For SDM meta-analytic procedures, we collected all available peak activation coordinates along with their corresponding statistical values (e.g., t-statistics, Z-scores, *p*-values). In studies that reported multiple tasks, participant groups, or training conditions, coordinates were extracted separately for each relevant contrast to ensure accurate modeling of condition-specific effects.

### 2.5. Study Characteristics

Across the 15 studies included in this meta-analysis [16,17,18,19,20,23,32,33,35,36,37,38,39,40,41], 541 participants were analyzed (mean age = 34.66 years; range = 7.8–61.6 years). On average, compliance with the training protocols was 92.57%. Training sessions lasted approximately 56.25 min each and occurred 3.37 times per week. Across studies, cognitive training programs comprised an average of 25.18 sessions, yielding a cumulative mean training dose of 1335 min. Detailed study characteristics are presented in Supplementary Table S5.

### 2.6. Multivariate Meta-Analysis

This multivariate meta-analysis quantified the behavioral effects of cognitive training on cognitive performance using the metafor and clubSandwich packages in RStudio (v1.4.1106). In contrast to traditional univariate models, the multivariate framework incorporates multiple effect sizes contributed by the same study, accounts for correlations among outcomes, and yields more precise and unbiased estimates [42]. Effect sizes were modeled hierarchically across two levels—individual outcomes (Level 1) nested within studies (Level 2)—with random effects specified at both levels to appropriately capture within-study dependencies.

Cognitive performance was defined as behavioral outcomes across core domains frequently examined in cognitive training research, including executive function (e.g., Stroop, task-switching, n-back, stop-signal tasks), memory (e.g., word-list recall, face–name encoding, associative memory, spatial delayed matching), attention (e.g., Posner cuing, visual search, divided attention), and processing speed (e.g., digit–symbol verification, simple visual detection). To estimate the overall behavioral impact of cognitive training, task-level effects were aggregated to form a standardized composite indicator of cognitive function, allowing comparisons across heterogeneous measurement scales [43].

Standardized mean differences (SMDs), expressed as Hedges’ g, were calculated to correct for small-sample bias and facilitate comparability across tasks [44]. Effect sizes were derived from pre–post differences or change scores and standardized using the pooled within-group standard deviation. For outcomes where lower scores indicated better performance (e.g., reaction time, error rates), effect signs were reversed so that positive values consistently reflected training-related improvement. Thus, larger Hedges’ g values correspond to greater gains in cognitive function.

The within-study correlation among outcomes was set to 0.5, following Cochrane Handbook guidance recommending this value as a pragmatic default when correlations cannot be directly extracted [45]. To assess robustness, sensitivity analyses were conducted by re-estimating all models using alternative correlation assumptions (0, 0.3, 0.8, and 1), in line with recent recommendations for multivariate meta-analytic practice [46].

### 2.7. SDM Meta-Analysis

This study used SDM (v5.15) to estimate task-related activation changes following cognitive training [47]. For each eligible study, peak coordinates, corresponding statistical values, thresholds, and sample sizes were extracted. Non-significant results were also recorded, and reported Z- or *p*-values were converted to t-statistics using the SDM converter. When studies did not provide sufficient statistical values, effect sizes were estimated from the available peak coordinates or significance thresholds following established SDM procedures.

Preprocessing followed standard SDM pipelines, including application of a 20 mm FWHM Gaussian kernel and 500 permutations. For studies reporting multiple contrasts within the same task or training condition, the combine image function was used to generate a single effect-size map representing the mean activation change and its variance across contrasts. Given the limited number of included studies, statistical inference employed a voxel-level threshold of uncorrected *p* < 0.0001, consistent with prior SDM research demonstrating that this threshold can approximate corrected results in small-sample neuroimaging meta-analyses [43,48]. Clusters were considered significant if they exhibited a peak SDM z-value ≥ 1 and a minimum spatial extent of ≥10 contiguous voxels [49]. Robustness of the findings was further evaluated using jackknife sensitivity analyses, in which the meta-analysis was iteratively recomputed while removing one study at a time. In addition, we conducted a population-restricted sensitivity analysis by re-running the meta-analysis after excluding studies enrolling cognitively unimpaired participants, to examine whether the main results were driven by these studies. We did not perform a formal subgroup meta-analysis for the cognitively unimpaired subgroup because the number of available studies was too small to support reliable subgroup inference and moderator testing [47].

### 2.8. Exploratory Analyses

To further elucidate how cognitive training modulates task-related brain activation, we conducted a series of exploratory analyses using activation values extracted from the significant clusters identified in the SDM meta-analysis. Specifically, we performed exploratory regression analyses to assess whether training parameters—sessions per week, session duration, intervention length, weekly duration, total number of sessions, and total training duration—as well as participant sex and age, were associated with training-related changes in neural activation. We additionally examined the association between cognitive improvement, indexed by Hedges’ g, and changes in brain activation to characterize brain–behavior relationships. A significance threshold of *p* < 0.05 was applied for all exploratory analyses.

### 2.9. Quality Assessment

To ensure the quality of the systematic review, we assessed the reporting quality of the included neuroimaging studies using criteria established in previous research [50]. This evaluation was based on a 17-item checklist encompassing key aspects such as participant characteristics, study design, data acquisition, preprocessing procedures, statistical analyses, and the reporting of conclusions. The full set of criteria is detailed in Supplementary Table S4.

## 3. Results

### 3.1. Meta-Analysis Results

#### 3.1.1. Overall Analysis Results

**Figure 2:**
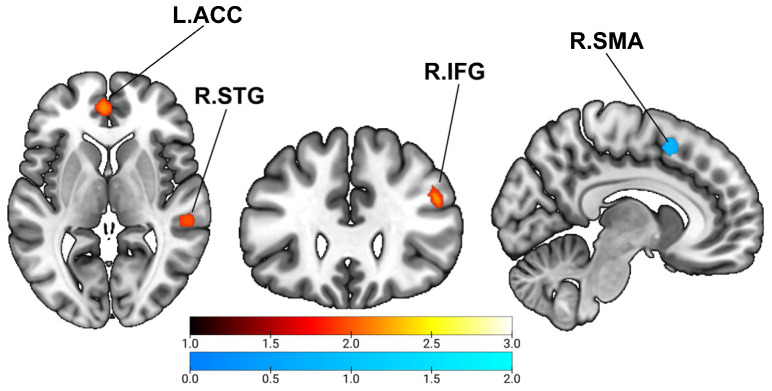
SDM meta-analysis results of task-related brain activation changes induced by cognitive training. Note: Brain regions identified by the SDM analysis were thresholded at uncorrected *p* < 0.0001, which is considered to provide a level of stringency comparable to corrected thresholds [43,48]. Clusters were considered significant if they exhibited a peak SDM z ≥ 1 and comprised ≥10 contiguous voxels. Red regions indicate increased activation, while blue regions indicate decreased activation induced by cognitive training. Warmer colors represent stronger positive effects, whereas cooler colors represent stronger negative effects. AL.ACC = Left anterior cingulate; R.IFG = Right inferior frontal gyrus; R.STG = Right superior temporal gyrus; R.SMA = Right supplementary motor area.

Cognitive training significantly improved cognitive performance relative to control conditions, yielding a pooled effect size of Hedges’ g = 0.485 (95% CI: 0.149–0.821; *t* = 2.924; *p* = 0.006). As summarized in Table 1 and illustrated in Figure 2, the overall SDM meta-analysis identified three significant clusters showing increased task-related activation following cognitive training compared with controls. These increases were primarily located in the left anterior cingulate cortex (L.ACC), right inferior frontal gyrus (R.IFG), and right superior temporal gyrus (R.STG). In contrast, one significant cluster demonstrated decreased activation in the right supplementary motor area (R.SMA).

#### 3.1.2. Exploratory Analyses Results

**Figure 3:**
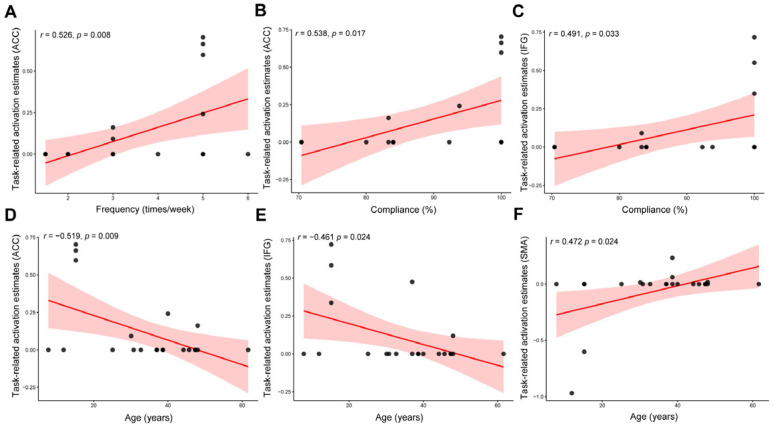
**Exploratory meta-regression analyses.** (**A**) Association between training frequency and task-related activation estimates in the ACC. (**B**) Association between training compliance and task-related activation estimates in the ACC. (**C**) Association between training compliance and task-related activation estimates in the IFG. (**D**) Association between age and task-related activation estimates in the ACC. (**E**) Association between age and task-related activation estimates in the IFG. (**F**) Association between ageand task-related activation estimates in the SMA. **Note:** Each dot represents one effect size; the solid line indicates the regression fit and the shaded area shows the 95% confidence interval. The meta-regression SDM value is derived from the proportion of studies that reported brain activation changes near the voxel; therefore, values for some studies may be 0 or close to 1 rather than lying close to the regression line [51]. ACC, anterior cingulate cortex; IFG, inferior frontal gyrus; SMA, supplementary motor area.

Exploratory regression results showed that training frequency was positively associated with increases in L.ACC activation following cognitive training (*r* = 0.526, *p* = 0.008; Figure 3A). Compliance was likewise significantly related to training-related changes in task-evoked activation in both the L.ACC (*r* = 0.538, *p* = 0.017; Figure 3B) and the R.IFG (*r* = 0.491, *p* = 0.033; Figure 3C). Age was also a significant predictor of neural changes, showing stronger increases in L.ACC (*r* = −0.519, *p* = 0.009; Figure 3D) and R.IFG activation (*r* = −0.461, *p* = 0.024; Figure 3E), as well as reduced activation in the R.SMA (*r* = 0.472, *p* = 0.024; Figure 3F).

**Figure 4:**
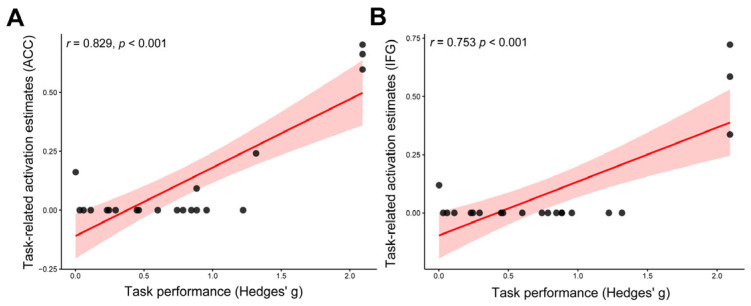
**Exploratory meta-regression analyses.** (**A**) Association between task performance and task-related activation estimates in the ACC. (**B**) Association between task performance and task-related activation estimates in the IFG. **Note:** Each point represents one effect size; the solid line indicates the regression fit and the shaded area shows the 95% confidence interval. **The meta-regression SDM value is derived from the proportion of studies that reported brain activation changes near the voxel, so it is expected that the values of some of the studies are at 0 or near + 1, instead of being close to the line [51].** L.ACC, left anterior cingulate cortex; R.IFG, right inferior frontal gyrus.

Cognitive task performance was positively associated with training-related increases in both L.ACC activation (*r* = 0.839, *p* < 0.001; Figure 4A) and R.IFG activation (*r* = 0.753, *p* < 0.001; Figure 4B). In contrast, session duration, intervention length, weekly training duration, total number of sessions, total training duration, and participant sex were not significantly associated with changes in neural activation or cognitive performance (all *p* > 0.05).

### 3.2. Reliability Analysis Results

As summarized in Supplementary Table S5, jackknife sensitivity analyses indicated that the task-related activation changes identified in the SDM meta-analysis were highly reproducible, emerging consistently across all leave-one-out iterations. In addition, a population-restricted sensitivity analysis excluding studies enrolling cognitively unimpaired participants yielded a highly similar pattern of results: cognitive training–related increases were primarily observed in the L.ACC, R.STG, and R.IFG, whereas one significant cluster showed decreased activation in the R.SMA (Supplementary Table S6 and Figure S1). Likewise, Supplementary Table S7 indicates that the behavioral meta-analytic findings were robust, with cognitive training effects on cognitive performance remaining stable across all tested assumptions regarding within-study correlations.

## 4. Discussion

This preregistered meta-analysis integrates multivariate behavioral and coordinate-based neuroimaging evidence to characterize cognitive and task-evoked neural findings associated with commercially available cognitive training programs. We observed a significant improvement in cognitive performance and convergent differences in task-related brain activation across studies.

The observed cognitive improvement aligns with prior evidence showing that commercial cognitive training can enhance cognitive function [21,22]. These cognitive benefits are consistent with theoretical accounts suggesting that repeated practice promotes more efficient cognitive control, attentional allocation and strategy optimization [25]. At the same time, this effect should be interpreted in the context of the broader debate regarding the limitations of cognitive training and transfer [14]. Compared with laboratory-based cognitive training that is often relatively single-domain and frequently uses the trained tasks themselves as outcome measures [29,30,31], commercial programs typically engage multiple domains, which may strengthen core components of cognitive control that generalize across tasks. Notably, most outcome measures included in the present meta-analysis were untrained tasks, suggesting that the benefits of commercial cognitive training are not limited to task-specific learning and may transfer to novel cognitive demands. Nevertheless, it is important to emphasize that the average effect may translate into relatively modest gains at the individual level and should therefore be interpreted with caution.

Beyond the behavioral improvements, the meta-analysis revealed a consistent pattern of training-related differences in task-evoked neural activation, with increased activation observed in the L.ACC and R.IFG, as well as in the R.STG. Specifically, the ACC is widely recognized for detecting performance-relevant demands and signaling the need for regulatory adjustments [52], whereas the IFG supports inhibitory control, attentional selection, and goal maintenance [53]. Training-related increases were also observed in the R.STG, a region implicated in perceptual integration and attentional reorienting [54]. Given its role in integrating sensory information and reallocating attention during task demands, increased STG activation may reflect heightened engagement of domain-general perceptual–attentional processes required by the experimental tasks [55], rather than processes directly driving cognitive improvement. Overall, this pattern is consistent with greater recruitment of task-related cognitive control (ACC/IFG) alongside enhanced perceptual integration and attentional reorienting (STG) during post-training task performance; however, activation increases may also reflect compensatory recruitment, greater task engagement, or task-specific strategy adjustments.

In contrast to these increases, training was associated with decreased activation in the R.SMA. The SMA plays an important role in motor preparation, action sequencing, and the allocation of attentional resources to response selection [56]. Reduced activation in this region may therefore be consistent with reduced motor–attentional demands during task performance following training, potentially reflecting changes in task strategy or more streamlined response selection [57]. Another possibility is that the decrease reflects a reduced dependence on compensatory motor recruitment, a pattern often observed when cognitive processes become more automatized or when higher-order control regions assume a larger share of task demands [58,59]. Taken together, increased activation in prefrontal control-related regions alongside decreased SMA activity may reflect a relative shift in task-related engagement between control and motor-related systems during task performance.

Training frequency and compliance were positively associated with increases in L.ACC and R.IFG activation. This pattern aligns with emerging dose–response frameworks that emphasize not only the amount of training but also the distribution or density of training bouts, suggesting that more frequent and consistently completed sessions may induce stronger neural adaptations—a principle highlighted in recent work on training density and brain health [60]. Age also emerged as a significant moderator. Older individuals exhibited smaller training-related increases in L.ACC and R.IFG activation, together with reduced decreases in R.SMA activation, indicating attenuated neural plasticity with advancing age. This pattern is consistent with evidence that aging is accompanied by declines in synaptic plasticity, reduced neurochemical responsivity, and diminished capacity to up-regulate task-relevant control regions in response to cognitive challenge [61]. Interpreting these age-dependent effects in relation to prominent cognitive-aging accounts, including the Compensation-Related Utilization of Neural Circuits Hypothesis (CRUNCH) and the Scaffolding Theory of Aging and Cognition (STAC) [62,63,64], suggests a coherent explanation for the observed moderation pattern. These frameworks posit that older adults increasingly recruit compensatory control resources and scaffolding mechanisms to sustain performance, while operating closer to functional capacity limits. Under this view, the smaller post-training increases in ACC/IFG activation may reflect a reduced capacity for further upregulation of control circuitry beyond an already elevated recruitment baseline. In parallel, the SMA is routinely implicated in response preparation and action sequencing, and post-training reductions in SMA activation are commonly interpreted as reflecting increased automatization and reduced response-related demands [57]. The attenuated SMA decreases in older adults may therefore indicate weaker efficiency-related downregulation, or a continued reliance on response-supporting resources under higher compensatory demand, which is compatible with compensatory maintenance interpretations derived from CRUNCH/STAC. Taken together, these findings suggest that inter-study variability in neural outcomes following commercially available cognitive training is shaped jointly by dose-related factors (frequency and compliance) and by age-related differences in neuroplastic capacity and compensatory recruitment.

Placing these findings within the broader landscape of prior reviews and meta-analyses helps to summarize general patterns of task-related activation change following cognitive training and to clarify the added value of the present focus on commercially available programs. First, meta-analytic work on working-memory training indicates that training-related neural changes are concentrated in frontoparietal control circuitry and select subcortical structures [29]. This pattern is broadly consistent with the present observation of training-related activation differences in key control nodes (e.g., ACC and IFG). Notably, because commercial programs are typically multi-module and involve a mixture of training ingredients, their most reproducible neural effects may preferentially manifest as modulation of domain-general control hubs rather than as a highly uniform replication of network-wide patterns reported for single-domain working-memory protocols. Second, a neuroimaging meta-analysis in older adults suggests that cognitive training is accompanied by increased activation in control-related prefrontal regions and potentially compensatory areas, and further indicates that age and brain–behavior relationships can shape how training effects are expressed [24]. In line with this, we observed control-related activation changes and identified age as a moderator of these effects, underscoring that training-related neurofunctional modulation in commercially deployed settings may be sensitive to age-related differences in plasticity. Third, a systematic review of aging and neurodegenerative disease trials emphasizes that post-training task-related activation can increase or decrease, plausibly reflecting complementary processes such as compensatory recruitment and improved processing efficiency [31]. Our co-occurring pattern of increased activation in control-related regions alongside decreased SMA activation is consistent with this mixed-direction literature and provides a useful empirical reference for interpreting both upregulation and downregulation in the context of commercial training. Finally, a meta-analysis comparing cognitive, physical, and meditative trainings reports training-specific modulation of fronto-medial regions implicated in control and performance monitoring, suggesting that plasticity within these systems may represent a shared neural substrate across intervention types [30]. Extending this evidence to a more ecologically deployed context, the present meta-analysis provides quantitative task-fMRI support that commercially available cognitive training engages control-related neural systems, thereby complementing prior syntheses and informing mechanistic interpretation of real-world digital training tools.

Several limitations should be acknowledged when interpreting these findings. First, the neuroimaging meta-analysis relied on studies reporting peak activation coordinates rather than full statistical maps, which may restrict the ability to capture distributed or network-level plasticity and introduce dependence on heterogeneous thresholding practices. Second, the number of eligible neuroimaging studies was relatively small, limiting the statistical power of the moderator analyses. Given that multiple moderators were examined in an exploratory manner with a small study-level sample, these moderator findings should be interpreted with caution. Third, most included trials lacked long-term follow-up assessments and connectivity analyses, limiting our ability to determine whether cognitive and neural changes persist beyond the immediate post-training period or to make strong inferences about underlying mechanisms. Fourth, the included trials exhibited a degree of heterogeneity in participant characteristics, intervention content and dose, and in-scanner task paradigms, all of which may influence task-related activation patterns. To address this variability, we conducted exploratory moderator analyses and identified meaningful moderation effects, thereby providing a more nuanced account of between-study differences.

Taken together, this meta-analysis provides convergent behavioral evidence of cognitive benefits associated with commercially available cognitive training programs and a quantitative synthesis of related task-evoked neural findings. Across studies, training was associated with convergent differences in task-related brain activation, including increased activation in frontal control-related regions and decreased activation in motor-related regions. In exploratory analyses, training intensity, adherence, and age were associated with between-study variability in these activation differences. By integrating behavioral and neuroimaging results, our findings identify convergent neural correlates that relate to cognitive gains at the study level and help characterize potential boundary conditions on training-related plasticity. These results inform the evaluation of real-world cognitive training tools by moving beyond behavioral outcomes to characterize the neural patterns that co-occur with cognitive improvement.

## Figures and Tables

**Table 1:** Brain regions with significant differences in task-related activation between cognitive training and control conditions in the overall analysis.

MNI Coordinate	SDM-Z	*p*	Voxels	Description
Cognitive training > controls				
−4, 46, −2	2.269	<0.0001	253	Left anterior cingulate
46, 26, 28	2.201	<0.0001	73	Right inferior frontal gyrus
54, −30, 4	1.988	<0.0001	64	Right superior temporal gyrus
Cognitive training < controls				
8, 12, 50	−1.258	<0.0001	50	Right supplementary motor area

Note: SDM-Z values reflect effect-size estimates derived from SDM meta-analysis. Positive values indicate greater activation for cognitive training relative to controls; negative values indicate reduced activation.

## Data Availability

All data used in this meta-analysis were extracted from previously published studies. No new data were created or analyzed in this study.

## Supplementary Materials

The following supporting information can be downloaded at: https://www.mdpi.com/article/10.3390/brainsci16010078/s1, Table S1: PRISMA 2020 Checklist; Table S2: Electronic database search; Table S3: Quality assessment checklist; Table S4: Participant, intervention, result and quality of included studies; Table S5: Results of the jackknife analysis in all included studies; Table S6: Population-restricted sensitivity analysis excluding studies enrolling cognitively unimpaired participants; Table S7: Sensitivity results of multivariate meta-analysis across assumed within-study correlations; Figure S1: SDM results after excluding studies enrolling cognitively unimpaired participants.

## Abbreviations

The following abbreviations are used in this manuscript:

CRUNCH	Compensation-Related Utilization of Neural Circuits Hypothesis
L.ACC	Left anterior cingulate cortex
PRISMA	Preferred Reporting Items for Systematic Reviews and Meta-Analyses
R.IFG	Right inferior frontal gyrus
R.SMA	Right supplementary motor area
R.STG	Right superior temporal gyrus
SDM	Seed-based d mapping
SMDs	Standardized mean differences
STAC	Scaffolding Theory of Aging and Cognition
