# Supplementary material for: Neural Correlates of Cognitive Gains Induced by Commercially Available Cognitive Training Programs: A Meta-Analysis of Neuroimaging Studies
Source: Brain Sci. 2026 Jan 6;16(1):78. doi: 10.3390/brainsci16010078 (PMC12839008; doi:10.3390/brainsci16010078)
Supplement: Supplementary file 1 [file brainsci-16-00078-s001.zip › brainsci-4042914-supplementary.pdf]

Supplementary Material

Table S1. PRISMA 2020 Checklist.

Section and Topic	Item	Checklist item	Location where item is reported
**TITLE**			
Title	1	Identify the report as a systematic review.	Manuscript Page 1
**ABSTRACT**			
Abstract	2	See the PRISMA 2020 for Abstracts checklist.	Manuscript Page 1
**INTRODUCTION**			
Rationale	3	Describe the rationale for the review in the context of existing knowledge.	Manuscript Page 2
Objectives	4	Provide an explicit statement of the objective(s) or question(s) the review addresses.	Manuscript Page 2
**METHODS**			
Eligibility criteria	5	Specify the inclusion and exclusion criteria for the review and how studies were grouped for the syntheses.	Manuscript Page 3
Information sources	6	Specify all databases, registers, websites, organisations, reference lists and other sources searched or consulted to identify studies. Specify the date when each source was last searched or consulted.	Manuscript Pages 2-3
Search strategy	7	Present the full search strategies for all databases, registers and websites, including any filters and limits used.	Manuscript Pages 2-3
Selection process	8	Specify the methods used to decide whether a study met the inclusion criteria of the review, including how many reviewers screened each record and each report retrieved, whether they worked independently, and if applicable, details of automation tools used in the process.	Manuscript Pages 2-3
Data collection process	9	Specify the methods used to collect data from reports, including how many reviewers collected data from each report, whether they worked independently, any processes for obtaining or confirming data from study investigators, and if applicable, details of automation tools used in the process.	Manuscript Pages 2-3
Data items	10a	List and define all outcomes for which data were sought. Specify whether all results that were compatible with each outcome domain in each study were sought (e.g. for all measures, time points, analyses), and if not, the methods used to decide which results to collect.	Manuscript Page 3
	10b	List and define all other variables for which data were sought (e.g. participant and intervention characteristics, funding sources). Describe any assumptions made about any missing or unclear information.	Manuscript Page 3
Study risk of bias assessment	11	Specify the methods used to assess risk of bias in the included studies, including details of the tool(s) used, how many reviewers assessed each study and whether they worked independently, and if applicable, details of automation tools used in the process.	Manuscript Pages 3-5
Effect measures	12	Specify for each outcome the effect measure(s) (e.g. risk ratio, mean difference) used in the synthesis or presentation of results.	Manuscript Pages 3-4
Synthesis methods	13a	Describe the processes used to decide which studies were eligible for each synthesis (e.g. tabulating the study intervention characteristics and comparing against the planned groups for each synthesis (item #5)).	Manuscript Pages 3-4
	13b	Describe any methods required to prepare the data for presentation or synthesis, such as handling of missing summary statistics, or data conversions.	Manuscript Pages 3-4
	13c	Describe any methods used to tabulate or visually display results of individual studies and syntheses.	Manuscript Pages 3-4
	13d	Describe any methods used to synthesize results and provide a rationale for the choice(s). If meta-analysis was performed, describe the model(s), method(s) to identify the presence and extent of statistical heterogeneity, and software package(s) used.	Manuscript Pages 3-4
	13e	Describe any methods used to explore possible causes of heterogeneity among study results (e.g. subgroup analysis, meta-regression).	Manuscript Pages 3-4

Section and Topic	Item	Checklist item	Location where item is reported
	13f	Describe any sensitivity analyses conducted to assess robustness of the synthesized results.	Manuscript Pages 3-4
Reporting bias assessment	14	Describe any methods used to assess risk of bias due to missing results in a synthesis (arising from reporting biases).	Manuscript Page 5
Certainty assessment	15	Describe any methods used to assess certainty (or confidence) in the body of evidence for an outcome.	Manuscript Page 5
**RESULTS**			
Study selection	16a	Describe the results of the search and selection process, from the number of records identified in the search to the number of studies included in the review, ideally using a flow diagram.	Manuscript Page 5
	16b	Cite studies that might appear to meet the inclusion criteria, but which were excluded, and explain why they were excluded.	Manuscript Page 5
Study characteristics	17	Cite each included study and present its characteristics.	Manuscript Page 6
Risk of bias in studies	18	Present assessments of risk of bias for each included study.	Manuscript Pages 19-22
Results of individual studies	19	For all outcomes, present, for each study: (a) summary statistics for each group (where appropriate) and (b) an effect estimates and its precision (e.g. confidence/credible interval), ideally using structured tables or plots.	Manuscript Pages 15-16
Results of syntheses	20a	For each synthesis, briefly summarise the characteristics and risk of bias among contributing studies.	Manuscript Pages 6-8
	20b	Present results of all statistical syntheses conducted. If meta-analysis was done, present for each the summary estimate and its precision (e.g. confidence/credible interval) and measures of statistical heterogeneity. If comparing groups, describe the direction of the effect.	Manuscript Pages 6-8
	20c	Present results of all investigations of possible causes of heterogeneity among study results.	Manuscript Pages 8-9
	20d	Present results of all sensitivity analyses conducted to assess the robustness of the synthesized results.	Manuscript Pages 8-9
Reporting biases	21	Present assessments of risk of bias due to missing results (arising from reporting biases) for each synthesis assessed.	Manuscript Pages 8-9
Certainty of evidence	22	Present assessments of certainty (or confidence) in the body of evidence for each outcome assessed.	Manuscript Pages 8-9
**DISCUSSION**			
Discussion	23a	Provide a general interpretation of the results in the context of other evidence.	Manuscript Pages 9-10
	23b	Discuss any limitations of the evidence included in the review.	Manuscript Pages 9-10
	23c	Discuss any limitations of the review processes used.	Manuscript Page 10
	23d	Discuss implications of the results for practice, policy, and future research.	Manuscript Page 10
**OTHER INFORMATION**			
Registration and protocol	24a	Provide registration information for the review, including register name and registration number, or state that the review was not registered.	Manuscript Page 2
	24b	Indicate where the review protocol can be accessed, or state that a protocol was not prepared.	Manuscript Page 2
	24c	Describe and explain any amendments to information provided at registration or in the protocol.	Manuscript Page 2
Support	25	Describe sources of financial or non-financial support for the review, and the role of the funders or sponsors in the review.	Manuscript Page 10
Competing interests	26	Declare any competing interests of review authors.	Manuscript Page 10
Availability of data, code and other materials	27	Report which of the following are publicly available and where they can be found: template data collection forms; data extracted from included studies; data used for all analyses; analytic code; any other materials used in the review.	Manuscript Page 10

Table S2. Electronic database search (most recent search date: 12 November 2024).

**Information source**	PubMed

**Search strategy**	("cognitive training"[tiab] OR "cognitive rehabilitation"[tiab] OR "cognitive therapy"[tiab] OR "neuropsychological therapy"[tiab] OR "cognitive intervention"[tiab] OR "executive function training"[tiab] OR "working memory training"[tiab] OR "memory training"[tiab] OR "attention training"[tiab] OR "processing speed training"[tiab] OR "brain training"[tiab] OR "computer-based cognitive training"[tiab] OR "virtual reality cognitive training"[tiab] OR "multitasking training"[tiab] OR "dual task training"[tiab] OR "cognitive flexibility training"[tiab] OR "inhibitory control training"[tiab] OR "switching training"[tiab] OR "n-back training"[tiab] OR "updating training"[tiab] OR "shifting training"[tiab] OR "flexibility training"[tiab] OR "multi-domain training"[tiab] OR "cognitive adaptation training"[tiab] OR "brain fitness"[tiab] OR "brain games"[tiab] OR "computerized cognitive training"[tiab]) AND ("fMRI"[tiab] OR "functional magnetic resonance imaging"[tiab] OR "PET"[tiab] OR "positron emission tomography"[tiab] OR "SPECT"[tiab] OR "single photon emission computed tomography"[tiab] OR "functional imaging"[tiab] OR "neuroimaging"[tiab] OR "neuroplasticity"[tiab] OR "neural activation"[tiab] OR "brain function"[tiab])
**No. of records**	700

**Information source**	MEDLINE
**Search strategy**	("cognitive training".ab,ti OR "cognitive rehabilitation".ab,ti OR "cognitive therapy".ab,ti OR "neuropsychological therapy".ab,ti OR "cognitive intervention".ab,ti OR "executive function training".ab,ti OR "working memory training".ab,ti OR "memory training".ab,ti OR "attention training".ab,ti OR "processing speed training".ab,ti OR "brain training".ab,ti OR "computer-based cognitive training".ab,ti OR "virtual reality cognitive training".ab,ti OR "multitasking training".ab,ti OR "dual task training".ab,ti OR "cognitive flexibility training".ab,ti OR "inhibitory control training".ab,ti OR "switching training".ab,ti OR "n-back training".ab,ti OR "updating training".ab,ti OR "shifting training".ab,ti OR "flexibility training".ab,ti OR "multi-domain training".ab,ti OR "cognitive adaptation training".ab,ti OR "brain fitness".ab,ti OR "brain games".ab,ti OR "computerized cognitive training".ab,ti) AND ("fMRI".ab,ti OR "functional magnetic resonance imaging".ab,ti OR "PET".ab,ti OR "positron emission tomography".ab,ti OR "SPECT".ab,ti OR "single photon emission computed tomography".ab,ti OR "functional imaging".ab,ti OR "neuroimaging".ab,ti OR "neuroplasticity".ab,ti OR "neural activation".ab,ti OR "brain function".ab,ti)
**No. of records**	165

**Information source**	PsycINFO
**Search strategy**	("cognitive training".ti,ab OR "cognitive rehabilitation".ti,ab OR "cognitive therapy".ti,ab OR "neuropsychological therapy".ti,ab OR "cognitive intervention".ti,ab OR "executive function training".ti,ab OR "working memory training".ti,ab OR "memory training".ti,ab OR "attention training".ti,ab OR "processing speed training".ti,ab OR "brain training".ti,ab OR "computer-based cognitive training".ti,ab OR "virtual reality cognitive training".ti,ab OR "multitasking training".ti,ab OR "dual task training".ti,ab OR "cognitive flexibility training".ti,ab OR "inhibitory control training".ti,ab OR "switching training".ti,ab OR "n-back training".ti,ab OR "updating training".ti,ab OR "shifting training".ti,ab OR "flexibility training".ti,ab OR "multi-domain training".ti,ab OR "cognitive adaptation training".ti,ab OR "brain fitness".ti,ab OR "brain games".ti,ab OR "computerized cognitive training".ti,ab) AND ("fMRI".ti,ab OR "functional magnetic resonance imaging".ti,ab OR "PET".ti,ab OR "positron emission tomography".ti,ab OR "SPECT".ti,ab OR "single photon emission computed tomography".ti,ab OR "functional imaging".ti,ab OR "neuroimaging".ti,ab OR "neuroplasticity".ti,ab OR "neural activation".ti,ab OR "brain function".ti,ab)
**No. of records**	1,025

**Information source**	Embase
**Search strategy**	("cognitive training".ti,ab OR "cognitive rehabilitation".ti,ab OR "cognitive therapy".ti,ab OR "neuropsychological therapy".ti,ab OR "cognitive intervention".ti,ab OR "executive function training".ti,ab OR "working memory training".ti,ab OR "memory training".ti,ab OR "attention training".ti,ab OR "processing speed training".ti,ab OR "brain training".ti,ab OR "computer-based cognitive training".ti,ab OR "virtual reality cognitive training".ti,ab OR "multitasking training".ti,ab OR

	"dual task training".ti,ab OR "cognitive flexibility training".ti,ab OR "inhibitory control training".ti,ab OR "switching training".ti,ab OR "n-back training".ti,ab OR "updating training".ti,ab OR "shifting training".ti,ab OR "flexibility training".ti,ab OR "multi-domain training".ti,ab OR "cognitive adaptation training".ti,ab OR "brain fitness".ti,ab OR "brain games".ti,ab OR "computerized cognitive training".ti,ab) AND ("fMRI".ti,ab OR "functional magnetic resonance imaging".ti,ab OR "PET".ti,ab OR "positron emission tomography".ti,ab OR "SPECT".ti,ab OR "single photon emission computed tomography".ti,ab OR "functional imaging".ti,ab OR "neuroimaging".ti,ab OR "neuroplasticity".ti,ab OR "neural activation".ti,ab OR "brain function".ti,ab)
**No. of records**	1,334

**Information source**	Web of science
**Search strategy**	("cognitive training" OR "cognitive rehabilitation" OR "cognitive therapy" OR "neuropsychological therapy" OR "cognitive intervention" OR "executive function training" OR "working memory training" OR "memory training" OR "attention training" OR "processing speed training" OR "brain training" OR "computer-based cognitive training" OR "virtual reality cognitive training" OR "multitasking training" OR "dual task training" OR "cognitive flexibility training" OR "inhibitory control training" OR "switching training" OR "n-back training" OR "updating training" OR "shifting training" OR "flexibility training" OR "multi-domain training" OR "cognitive adaptation training" OR "brain fitness" OR "brain games" OR "computerized cognitive training") AND ("fMRI" OR "functional magnetic resonance imaging" OR "PET" OR "positron emission tomography" OR "SPECT" OR "single photon emission computed tomography" OR "functional imaging" OR "neuroimaging" OR "neuroplasticity" OR "neural activation" OR "brain function")
**No. of records**	1,435

Table S3. Quality assessment checklist.

**Subjects**
1. The sample size was appropriate
2. Subjects were evaluated prospectively, demographic data were reported (age (mean and SD/range), sex, and handedness), and psychiatric and medical illnesses were excluded
3. If any subject was scanned but then rejected from the analysis, withdrawals from the study were explained
**Methods for tasks**
4. All participants went through a training session outside the scanner
5. The experimental design was meticulously detailed to enable replication, including the number of blocks or trials per participant, the duration of each trial, the inter-stimulus interval, and whether the design was block-based or event-related.
6. The stimuli and the number of repetitions were sufficient and clearly described
7. If applicable, the baseline condition was defined as almost the same as the task condition.
**Methods for image acquisition and statistical analysis**
8. MRI slice thickness ≤ 3 mm
9. 3T MRI was used
10. The imaging technique used for data acquisition was clearly described so that it could be reproduced (e.g., MRI system used, field strength, pulse sequence type, number of volumes per session, field of view, matrix size, slice thickness, interslice skip, acquisition orientation, TE/TR/flip angle)
11. Preprocessing operations were clearly described and detailed so that they could be reproduced (e.g., software used, order of preprocessing operations, slice-timing, motion correction, coregistration and normalization (linear/affine or nonlinear), smoothing)
12. Adjustments were made for multiple statistical comparisons
13. Appropriate design and/or analytical methods to control confounding
14. Appropriate use of statistics for primary analysis effect (excluding control of confounders)
**Results, conclusions, and conflicts of interest**
15. Statistical parameters for significant and important nonsignificant differences were provided
16. Conclusions were consistent with the results obtained and the limitations were discussed
17. Declarations of conflicts of interest or identification of funding sources

Note: Score 0/0.5/1 for each item (0.5 points were given for criteria partially met).

Table S4. Participant, intervention, result and quality of included studies.

No.	Study (year)	Cognitive status	Intervention group (n)	Intervention details	Control group (n)	In-scan tasks	Age (years)	Female (%)	Quality
1	Bor et al., 2011	Cognitively unimpaired	Rehacom® (8)	Sessions per week: 2<br>Session duration: 120 min<br>Intervention length: 7 weeks<br>Weekly duration: 240 min<br>Total sessions: 14<br>Total training duration: 1680 min	Usual care (9)	N-back	30.76	28.6	16
2	Campbell et al., 2016	Cognitively impaired	RehaCom® (19)	Sessions per week: 3<br>Session duration: 45 min<br>Intervention length: 6 weeks<br>Weekly duration: 135 min<br>Total sessions: 18<br>Total training duration: 810 min	Video watching (19)	N-back	47.37	34.2	17
3	Cerasa et al., 2012	Cognitively impaired	RehaCom® (12)	Sessions per week: 2<br>Session duration: 60 min<br>Intervention length: 6 weeks<br>Weekly duration: 120 min<br>Total sessions: 12<br>Total training duration: 720 min	Placebo cognitive training (11)	PVSAT	32.7	46.2	15.5
4	Conklin et al., 2015	Cognitively impaired	Cogmed® (30)	Sessions per week: 4<br>Session duration: 37.5 min<br>Intervention length: 7 weeks<br>Weekly duration: 150 min<br>Total sessions: 28<br>Total training duration: 1050 min	Wait-list (30)	VSWMT	11.9	47	14.5
5	Filippi et al., 2012	Cognitively impaired	RehaCom® (10)	Sessions per week: 3<br>Session duration: 60 min<br>Intervention length: 12 weeks<br>Weekly duration: 180 min<br>Total sessions: 36<br>Total training duration: 2160 min	Usual care (10)	Stroop	45.7	100	16.5
6	Gunning et al., 2021	Cognitively impaired	EVO® (34)	Sessions per week: 6	None	T1: Stroop	61.6	71.2	15

				Session duration: 22.5 min<br>Intervention length: 4 weeks<br>Weekly duration: 135 min<br>Total sessions: 24<br>Total training duration: 550 min		T2: Flanker			
7	Haut et al., 2010	Cognitively impaired	CogPack® (10)	Sessions per week: 1-2<br>Session duration: 60 min<br>Intervention length: 5 weeks<br>Weekly duration: 90 min<br>Total sessions: 8<br>Total training duration: 480 min	Social skills training (20)	T1: N-back<br>T2: Lexical decision task	38.63	41	17
8	Kable et al., 2017	Cognitively unimpaired	Lumosity® (64)	Sessions per week: 5<br>Session duration: 30 min<br>Intervention length: 10 weeks<br>Weekly duration: 150 min<br>Total sessions: 50<br>Total training duration: 1500 min	Video game (64)	Delay discounting task	25.1	44.5	14
9	Kelly et al., 2020	Cognitively unimpaired	Cogmed® (23)	Sessions per week: 5<br>Session duration: 45 min<br>Intervention length: 6 weeks<br>Weekly duration: 225 min<br>Total sessions: 30<br>Total training duration: 1350 min	None	N-back	7.8	55.6	16
10	Meusel et al., 2013	Cognitively impaired	PSSCogRehab® (23)	Sessions per week: 3<br>Session duration: 60 min<br>Intervention length: 10 weeks<br>Weekly duration: 180 min<br>Total sessions: 30<br>Total training duration: 1800 min	None	T1: Encoding–retrieval task<br>T2: N-back	48	78.94	15
11	Ott et al., 2021	Cognitively impaired	Happy Neuron Pro® (26)	Sessions per week: 2<br>Session duration: 120 min<br>Intervention length: 10 weeks<br>Weekly duration: 240 min<br>Total sessions: 20<br>Total training duration: 2400 min	Unstructured conversation (19)	N-back	37	78	17
12	Ramsay et al., 2017	Cognitively unimpaired	CogRehab® (15)	Sessions per week: 3<br>Session duration: 60 min	Computer skills training (12)	N-back	44.18	NR	14.5

				Intervention length: 16 weeks<br>Weekly duration: 180 min<br>Total sessions: 48<br>Total training duration: 2880 min					
13	Subramaniam et al., 2012	Cognitively impaired	Brain Fitness Program® (14)	Sessions per week: 5<br>Session duration: 60 min<br>Intervention length: 8 weeks<br>Weekly duration: 300 min<br>Total sessions: 40<br>Total training duration: 2400 min	None	Reality monitoring task	40	16.13	16.5
14	Kim et al., 2009	Cognitively impaired	ComCog® (10)	Sessions per week: 3<br>Session duration: 30 min<br>Intervention length: 4 weeks<br>Weekly duration: 90 min<br>Total sessions: 12<br>Total training duration: 360 min	None	Visual attention task	30.1	30	15.5
15	Stevens et al., 2016	Cognitively impaired	Cogmed® (18)	Sessions per week: 5<br>Session duration: 30 min<br>Intervention length: 5 weeks<br>Weekly duration: 150 min<br>Total sessions: 25<br>Total training duration: 750 min	None	WISC	15.2	33.29	14

Abbreviations: n = number of participants included; T = task; NR = not reported; PVSAT = Paced Visual Serial Addition Test; VSWMT = Visual–Spatial Working Memory Task; WISC = Wechsler Intelligence Scale for Children

Table S5. Results of the jackknife analysis in all included studies.

Jackknife sensitivity analysis, discarded study	L.ACC	R.IFG	R.STG	R.SMA
Bor et al., 2011	Yes	Yes	Yes	Yes
Campbell et al., 2016	Yes	Yes	Yes	Yes
Cerasa et al., 2013	Yes	Yes	Yes	Yes
Conklin et al., 2015	Yes	Yes	Yes	Yes
Filippi et al., 2012	Yes	Yes	Yes	Yes
Gunning et al., 2021	Yes	Yes	Yes	Yes
Haut et al., 2010	Yes	Yes	Yes	Yes
Kable et al., 2017	Yes	Yes	Yes	Yes
Kelly et al., 2020	Yes	Yes	Yes	Yes
Meusel et al., 2013	Yes	Yes	Yes	Yes
Ott et al., 2021	Yes	Yes	Yes	Yes
Ramsay et al., 2017	Yes	Yes	Yes	Yes
Subramaniam et al., 2012	Yes	Yes	Yes	Yes
Kim et al., 2009	Yes	Yes	Yes	Yes
Stevens et al., 2016	Yes	Yes	Yes	Yes

Abbreviations: L.ACC = Left anterior cingulate; R.IFG = Right inferior frontal gyrus; R.STG = Right superior temporal gyrus; R.SMA = Right supplementary motor area.

Table S6. Population-restricted sensitivity analysis excluding studies enrolling cognitively unimpaired participants.

MNI coordinate	SDM-Z	*p*	Voxels	Description
Cognitive training > controls				
-4, 46, -2	2.548	< 0.0001	289	Left anterior cingulate
54, -30, 4	2.225	< 0.0001	88	Right superior temporal gyrus
46, 26, 28	2.436	< 0.0001	76	Right inferior frontal gyrus
Cognitive training < controls				
8, 12, 50	-1.321	< 0.0001	50	Right supplementary motor area

Note: Population-restricted sensitivity analysis was conducted by excluding studies enrolling cognitively unimpaired participants and re-running the SDM meta-analysis using the same preprocessing and statistical settings as the primary analysis. The resulting pattern of activation changes was broadly consistent with the primary analysis. SDM-Z values reflect effect-size estimates derived from SDM meta-analysis. Positive values indicate greater activation for cognitive training relative to controls; negative values indicate reduced activation.

.

Table S7. Sensitivity results of multivariate meta-analysis across assumed within-study correlations.

r	**Hedges' g**	**SE**	**CI**	***p***	***t***
0	0.487	0.168	0.146, 0.828	0.006	2.887
0.3	0.487	0.167	0.148, 0.826	0.005	2.907
0.8	0.481	0.163	0.152, 0.812	0.005	2.951
1	0.478	0.161	0.153, 0.804	0.005	2.972

Figure S1. SDM results after excluding studies enrolling cognitively unimpaired participants.

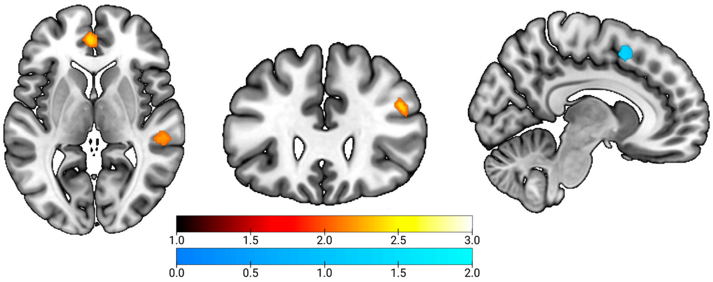

Note: Population-restricted sensitivity analysis was performed by excluding studies enrolling cognitively unimpaired participants and re-running the SDM meta-analysis using the same preprocessing and statistical settings as the primary analysis. The spatial pattern of training-related activation changes was broadly consistent with the primary analysis. Red regions indicate increased activation, while blue regions indicate decreased activation induced by cognitive training. Warmer colors represent stronger positive effects, whereas cooler colors represent stronger negative effects.
